# Development of an Attenuated Tat Protein as a Highly-effective Agent to Specifically Activate HIV-1 Latency

**DOI:** 10.1038/mt.2016.117

**Published:** 2016-07-19

**Authors:** Guannan Geng, Bingfeng Liu, Cancan Chen, Kang Wu, Jun Liu, Yijun Zhang, Ting Pan, Jun Li, Yue Yin, Junsong Zhang, Feng Huang, Fei Yu, Jingliang Chen, Xiancai Ma, Jie Zhou, Ersheng Kuang, Chao Liu, Weiping Cai, Hui Zhang

**Affiliations:** 1 Institute of Human Virology, Zhongshan School of Medicine, Sun Yat-Sen University, Guangzhou, China; 2 Key Laboratory of Tropical Disease Control of Ministry of Education, Zhongshan School of Medicine, Sun Yat-Sen University, Guangzhou, China; 3 Department of Infectious Diseases, Guangzhou 8th People's Hospital, Guangzhou, China

## Abstract

Although combined antiretroviral therapy (cART) successfully decreases plasma viremia to undetectable levels, the complete eradication of human immunodeficiency virus type 1 (HIV-1) remains impractical because of the existence of a viral reservoir, mainly in resting memory CD4^+^ T cells. Various cytokines, protein kinase C activators, and histone deacetylase inhibitors (HDACi) have been used as latency-reversing agents (LRAs), but their unacceptable side effects or low efficiencies limit their clinical use. Here, by a mutation accumulation strategy, we generated an attenuated HIV-1 Tat protein named Tat-R5M4, which has significantly reduced cytotoxicity and immunogenicity, yet retaining potent transactivation and membrane-penetration activity. Combined with HDACi, Tat-R5M4 activates highly genetically diverse and replication-competent viruses from resting CD4^+^ T lymphocytes isolated from HIV-1-infected individuals receiving suppressive cART. Thus, Tat-R5M4 has promising potential as a safe, efficient, and specific LRA in HIV-1 treatment.

## Introduction

Latent infection of human immunodeficiency virus type 1 (HIV-1) in resting CD4^+^ T lymphocytes is the major obstacle in virus eradication after HIV-1-infected individuals receive suppressive combined antiretroviral therapy (cART).^1,2,3,4,5^ The deficiency of transcriptional factors such as NF-kB or NFAT,^6,7^ the condensed chromatin structure, and epigenetic suppression could contribute to maintaining HIV-1 latency.^6,7,8,9,10,11^ The lack of viral regulatory protein Tat also plays an important role.^12^ In addition, a cluster of miRNAs including miR-28, miR-125b, miR-150, miR-223, and miR-382, which are enriched in resting CD4+ T lymphocytes, target the 3′-UTR of HIV-1 mRNA to inhibit the translation of viral proteins, are also involved in HIV-1 latency.^13^ Recently, the “shock and kill” strategy has been extensively discussed for the elimination of the viral reservoir.^14,15^ By driving latent viruses out of their hiding places, latency activators can expose infected cells under immune surveillance and lead to their eradication. However, there is no reliable method to effectively activate HIV-1 latency at present. Many general lymphocyte activators (*e.g.*, anti-CD3 monoclonal antibody, interleukin-2 (IL-2), and IL-7), non-specific transcription activators such as protein kinase C activators (*e.g.*, prostratin and bryostatin-1), and histone deacetylase inhibitors (HDACi) (*e.g.*, valproic acid and suberoylanilide hydroxamic acid (SAHA)) have been used as latency-reversing agents (LRAs) *ex vivo*. Some of them have even been tested in clinical trials.^16,17,18,19,20,21,22,23^ Unfortunately, none of them has been proved to effectively decrease the viral reservoir *in vivo*. Apparently, the development of more special and effective agents to activate viral latency is of great significance.

HIV-1 Tat is a 14–15-kDa early-phase protein of viral transcription. The two effective forms of Tat protein are an 86-amino acid (Tat-86) protein and a 101-amino acid (Tat-101) protein with an extra C-terminal domain. Both forms of Tat exist *in vivo*, with the 101-amino acid form being more immunogenic and inducing a stronger immune reaction.^24,25^ Tat specifically activates the HIV-1 promoter by interacting with transactivation response elements and recruiting some important transcriptional factors such as the P-TEFb complex, which contains CDK9 and cyclin T1. CDK9 kinase therefore hyper-phosphorylates the C-terminal domain of RNA polymerase II, leading to a significant increase in transcription efficiency. Tat also recruits CBP/P300 and PCAF to promote histone acetylation. In addition, Tat penetrates the cellular membrane easily because it contains a cell-penetrating peptide, which has widely been used to mediate the entry of various proteins from the extracellular space into the cytoplasm.^26,27^ However, Tat causes severe cytopathic effects, including apoptosis, and contributes to HIV-1 pathogenesis.^28,29,30,31^ Because of the specificity and efficiency of Tat to activate HIV-1 transcription and the proven safety of bioactive recombinant Tat protein in several clinical trials for vaccine development, it is possible to develop a mutated Tat protein as a novel HIV-1 latency activator by decreasing its cytotoxicity and immunogenicity.^32,33,34,35,36^ In this study, we employed multiple mutations and explored various combinations of mutants and have developed a recombinant mutated Tat protein for the effective activation of HIV-1 latency.

## Results

### Mutation strategy of HIV-1 Tat-86

**Figure 1:**
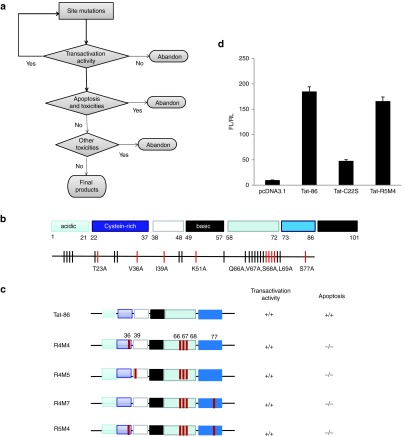
**Mutation strategy and final products**. (**a**) Mutation strategy and (**b**) positions of site mutations. Amino acids in mutation sites are all turned to Ala. Black sites stand for mutations decreasing the transactivation activity, and red sites stand for mutants remaining most of transactivation activity. (**c**) Final products after several rounds of combined site mutations. (**d**) Transactivation activity of Tat-R5M4 tested in TZM-bl cells by transfection of Tat-R5M4-exressing plasmids. FL/RL ratio is assumed to be the ratio of Photinus luciferase RLU to Renilla luciferase RLU.

A detailed structure–function association of HIV-1 Tat has been demonstrated in an earlier study.^37^ As the region containing amino acids 87–101 in Tat-101 is immunogenic and is not necessary for transactivation, and Tat-72 from the first exon of Tat mRNA has a much lower transactivation activity (**Supplementary Figure S1a**), we started our investigation with the Tat-86. Given that many regions of Tat have already been studied by various site mutations.^37,38,39,40^ We initially focused on the region of amino acids 60–72, which seems to be unrelated to transactivation activity. We also focused on sites whose mutations do not lead to the loss of transactivation ability or are not within the well-known essential domains for transactivation.^37^ Several domains that are important for Tat-mediated cytopathic effects have especially been subjected to point mutations.^41,42^ The amino acids in the mutation sites were replaced by Ala (GCA). By following these criteria, a series of Tat mutants were constructed by site mutations. All mutants were then transfected into TZM-b1 cells to examine the transactivation activity, while the cytopathic effects were also examined for these mutations, which still had potent transactivation activity (**Figure 1a**, **b**). By examining the effects of a series of mutations, we identified that MT23A, MV36A, MI39A, MK51A, MQ66A, MV67A, MS68A, ML69A and retained more than 70% of transactivation activity (**Supplementary Table S1**). We then combined these mutations in different groups and further tested the transactivation activities. In addition, random mutations and combinations with other mutations were conducted, but the results were not promising (**Supplementary Figure S1b–g**). After several rounds of combined mutations and repeated examinations for transactivation and cytopathic effects (**Supplementary Tables S1 and S2**), four candidates were obtained: Tat-R4M4, Tat-R4M5, Tat-R4M7, and Tat-R5M4 (**Supplementary Figures S1-S3**). For the maximum reduction of the cytotoxicity of Tat protein, we chose Tat-R5M4 for the following studies (**Figure 1c**). In comparison with wild-type Tat-86, we found that Tat-R5M4 has a similar ability to increase the expression of LTR-driven luciferase in TZM-bl cells through plasmid transfection (**Figure 1d**).

### Mutant R5M4 can efficiently activate HIV-1 LTR *in vitro* and has a reduced ability to induce apoptosis

**Figure 2:**
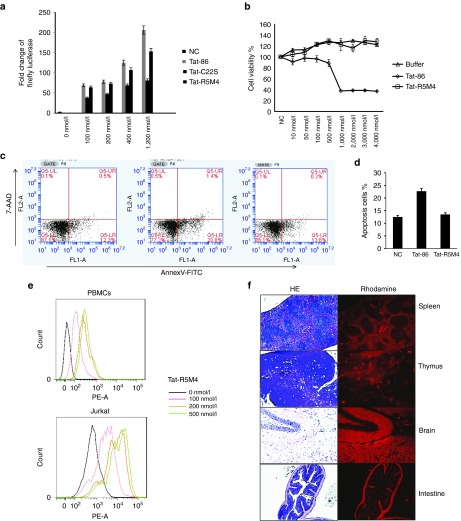
**The analysis of various Tat-R5M4 characteristics**. (**a**) The transactivation activity of Tat-R5M4 protein compared with Tat-86 and Tat-C22S mutant. After J-Lat cells were treated with purified Tat-86 and Tat-R5M4 at various concentrations for 48 hours, the luciferase activity was analyzed. For determining the cell toxicity of Tat-R5M4, Jurkat cells were treated with Tat-86 or Tat-R5M4, (**b**) cell viability was measured with MTS (3-[4,5-diethylthiazol-2-...(4-sulfo phenyl)-2H-etrazolium), inner salt) assay. After the treatments of various reagents for 2 days, the cell titer 96 aqueous one solution reagent (Promega) was added. The cell viability was then determined by measuring the absorbance at 493 nm; (**c**) apoptosis analysis. The primary CD4^+^ T cells were initially stained with Annexin V-PE and 7AAD, then analyzed by FACS, and (**d**) the results from three independent experiments were shown (mean ± SEM). (**e**) For determining the transmembrane activity of Tat-R5M4, the human peripheral blood mononuclear cells and Jurkat cells were treated with rhodamine-labeled Tat-R5M4 for 4 hours, and then analyzed by FACS to examine the transmembrane activity of Tat-R5M4. (**f**) For determining the delivery capability of Tat-R5M4 *in vivo*, Tat-R5M4 was labeled with rhodamine and intravenously injected into 4-week-old BABL/c mice. The multiple organs including spleen, thymus, and brain tissues were dissected, prepared with cryostat sections, and observed by fluorescence microscopy.

Subsequently, the recombinant proteins of both wild-type Tat-86 and Tat-R5M4 were expressed in *Escherichia coli* and purified (**Supplementary Figure S2**). Significant dose-dependent transactivation activity was observed when the purified recombinant proteins were directly added into the culture medium of TZM-bl cells, as well as a HIV-1 latently-infected cell line named J-Lat cells^43^ (**Figure 2a**, **Supplementary Figure S4**). These results indicated that Tat-R5M4 maintained a similar transactivation activity as that of wild-type Tat protein. Conversely, to examine the cytopathic effect of Tat-R4M5 protein, its cytotoxicity and ability to induce the apoptosis of uninfected CD4+ T cells were examined. Compared with wild-type Tat, Tat-R5M4 showed a significant reduction in total cell toxicity and ability to induce apoptosis (**Figure 2c**, **d**).

### *In vitro* and *in vivo* penetration capability of Tat-R5M4

To investigate the ability of Tat-R5M4 protein to penetrate the cellular membrane, Jurkat cells and freshly prepared human peripheral blood mononuclear cells were treated with rhodamine-labeled Tat-R5M4 and were analyzed by Fluorescence Activated Cell Sorting (FACS). The result showed 100% entry of Tat-R5M4 into the cells (**Figure 2e**). Fluorescence microscopy revealed the abundance of Tat-R5M4 within cells to be dose-dependent (**Supplementary Figure S5**). To further study the intracellular localization of Tat-R5M4, rhodamine-labeled protein was added into TZM-bl cell culture. Fluorescence observation showed that most Tat-R5M4 proteins were localized in the cytoplasm, and a small amount of protein localized in the nucleus suggested the high transactivation efficiency of Tat-R5M4 (**Supplementary Figure S6**). To access the delivery ability of Tat-R5M4 *in vivo*, Tat-R5M4 was labeled with NHS-rhodamine and intravenously injected into BALB/c mice. CD4^+^ T lymphocytes from both the spleen and bone marrow revealed strong fluorescence signals (**Supplementary Figure S7a**). Simultaneously, the tissues from several organs, including the spleen, thymus, intestine, and brain, were also dissected and subjected to fluorescence microscopy analysis. Strong signals were found in all tissue sections (**Figure 2f**, **Supplementary Figure S7b**), indicating that Tat-R5M4 can effectively cross the blood–brain barrier and blood–thymus barrier and penetrate into the tissues that are important for the formation of HIV-1 reservoir.^44,45,46,47^

### Tat-R5M4 activates latently infected cells in an *in vitro* latency model

**Figure 3:**
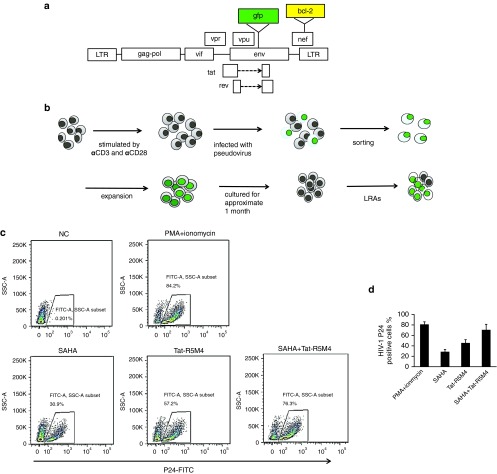
**Generation of *in vitro* latency model based on primary CD4**^**+**^ **T cells and reactivation activity of Tat-R5M4.** (**a**) The constructs used for packaging Bcl-2-expressing HIV-1/VSV-G pseudoviruses. The *Bcl-2* gene was inserted into the *nef* region of pNL4-3-*env-gfp*. (**b**) Strategy to generate *in vitro* latency model with primary CD4^**+**^ T-lymphocytes. Human primary CD4^+^ T cells were isolated from HIV-1 negative donors and activated with anti-CD3 and anti-CD28 antibodies for 48 hours. The cells were then infected with HIV-1-▵E-*gfp*/VSV-G pseudoviruses. The GFP-positive cells were sorted out and expanded with anti-CD3 and anti-CD28 antibodies. After cultured for approximate 1 month and the cells eventually turned to the resting state, various reagents were added to activate the expression of HIV-1 proteins. (**c**) The latently-infected CD4^**+**^ T-lymphocytes were activated with 25 ng/ml phorbol myristate acetate, 1 mg/ml ionomycin, 335 nmol/l suberoylanilide hydroxamic acid (SAHA), 1 µmol/l Tat-R5M4, or 335 nmol/l SAHA plus 1 µmol/l Tat-R5M4. At 24 hours after activation, the cells were stained with anti-p24-FITC and analyzed by FACS, and (**d**) the results from three independent experiments were shown (mean ± SEM).

The transduction of *Bcl-2* into primary CD4^+^ T cells can maintain the survival of resting memory CD4^+^ T cells.^48^ To investigate the ability of Tat-R5M4 to activate latently infected cells *in vitro*, we adapted the latency model based on primary CD4^+^ T cells infected with a modified *env*-deleted proviral construct harboring the *Bcl-2* gene in the *nef* region (**Figure 3a**). The freshly activated CD4^+^ T lymphocytes were infected with HIV-1/VSV pseudotyped viruses. Bcl-2 was expressed well and did not reduce the ratio of apoptosis after infection (**Supplementary Figure S8**). After all the cells harboring the integrated proviruses went into the resting state (**Supplementary Figure S8**), GFP-negative cells were isolated and subjected to reactivation by various reagents (**Figure 3b**, **Supplementary Figure S8**). Phorbol myristate acetate (PMA)/ionomycin, SAHA, and Tat-R5M4 were able to activate HIV-1 expression at 84, 31, and 57% respectively. The combination of Tat-R5M4 and SAHA showed 76% activation efficiency, which was much better than the individual treatments (**Figure 3c**).

### Tat-R5M4 significantly induces replication-competent and genetically diverse viruses from the resting CD4^+^ T lymphocytes isolated from HIV-1-infected individuals treated with ART

**Figure 4:**
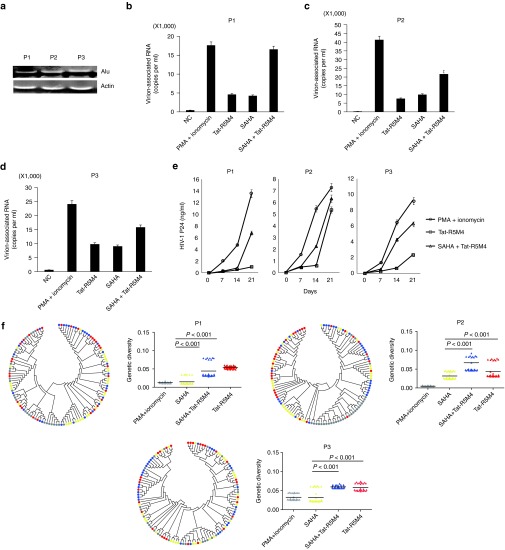
**Activation of the latently-infected CD4**^**+**^ **T-lymphocytes from HIV-1-infected individuals receiving suppressive cART by Tat-R5M4**. (**a**) Proviruses in CD4^+^ T-lymphocytes isolated from HIV-1-infected individuals were detected by Alu-PCR. (**b–d**) The CD4^+^ T-lymphocytes isolated from HIV-1-infected individuals were cultured in the RPMI1640 conditioned medium and activated with phorbol myristate acetate (PMA) plus ionomycin, suberoylanilide hydroxamic acid (SAHA), Tat-R5M4, or SAHA plus Tat-R5M4. After 48 hours, the viral particles in supernatant were harvested and viral RNA was extracted and quantitatively analyzed by real-time reverse transcription-PCR. (**e**) The CD4^+^ T-lymphocytes were exposed to 200Gy X-ray irradiation and then co-cultured with the freshly-activated CD4^**+**^ T-lymphocytes from HIV-1-negative donors. The viral replication was monitored with the continuous detection of HIV-1 p24 antigen in the supernatant. (**f**) Genetic diversity analysis. HIV-1 strains were sampled from three virally activated subjects treated by PMA plus ionomycin (gray), SAHA (yellow), SAHA plus Tat (blue) and Tat (red) respectively, and 30 clones were sequenced in each case. Right panel: each triangle represents the genetic distance between one given clone and the relevant entire population, and the horizontal bar indicates the mean. Left panel: the bootstrap consensus trees were constructed based on HIV-1 sequences obtained from the corresponding patients annotated in the right panel. The two-tailed Mann-Whitney *U*-test was used to compare the genetic diversities between different groups.

Resting CD4^+^ T lymphocytes were isolated from 3 HIV-1-infected individuals receiving suppressive cART and proved to be provirus-positive by Alu-PCR (**Figure 4a**). HIV-1 viral production could be stimulated from these resting CD4^+^ T cells by anti-CD3 and anti-CD28 (**Supplementary Figure S9a**). These cells were then treated with PMA/ionomycin, SAHA, Tat-R5M4, or Tat-R5M4 plus SAHA. While SAHA increased the production of HIV-1 viral particles by approximately 1.5–2-folds, Tat-R5M4 showed higher activity than SAHA. Again, the combination of Tat-R5M4 and SAHA showed a significantly higher activity compared to the separate treatments (**Figure 4b**–**d**). In contrast, the glutaraldehyde-treated Tat loss the transactivation activity (**Supplementary Figure S9b–d**). To examine whether the activated viruses were replication-competent, the activated cells from HIV-1-negative donors were cocultured with activated CD4^+^ T cells from HIV-1-infected individuals receiving suppressive ART. The consistent production of p24 antigen in the supernatant showed that the viruses activated by Tat-R5M4 were replication-competent (**Figure 4e**). Interestingly, genetic diversity analysis of virion-associated RNA in the supernatant of treated CD4^+^ T lymphocytes before coculture showed that Tat-R5M4 or Tat-R5M4 plus SAHA induced more HIV-1 quasispecies than SAHA or even the PMA/ionomycin combination did. This finding indicated that Tat-R5M4 was able to activate a larger number of HIV-1 proviruses at different locations, which could arise from the various dominant viral quasispecies at different time points of disease development to randomly integrate into host chromosomal DNA (**Figure 4f**).

### Tat-R5M4 has few side effects and low immunogenicity

**Figure 5:**
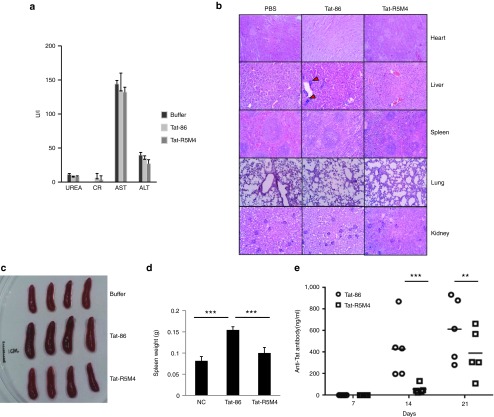
**Analysis of acute toxicity and immunogenicity for Tat-R5M4**. (**a**) The female Balb/c mice weighing 19 ± 1 g were intravenously injected with Tat-R5M4 at 40 mg/kg. The blood samples were collected at day 7 and subjected to the detections of blood glutamate-oxaloacetate transaminase (ALT), glutamate-pyruvate transaminase (AST), blood urea nitrogen (UREA), creatine (CR). (**b**) Mice were sacrificed after 7 days; their multiple organs were fixed in 4% formaldehyde for hematoxylin and eosin staining. The red triangles indicate the inflammatory cell infiltration. (**c**) After intravenous injection of proteins, the sizes of spleens from each group were measured, and (**d**) the weight changes induced by Tat-86 and Tat-R5M4 were measured and the mean ± SEM of three independent experiments are shown in panels **b–e**, The unpaired *t*-tests were used. **P < 0.01, ***P < 0.001. (**e**) The concentrations of anti-Tat antibodies induced by Tat-86 and Tat-R5M4. The 4-week-old female BABL/c mice were immunized subcutaneously with 1 μg Tat-86 or Tat-R5M4 protein (*n* = 5 per group) in combination with the complete Freund's adjuvant (Sigma) on day 0, and boosted three times with antigens in combination with the incomplete Freund's adjuvant (IFA) (Sigma). The concentration of anti-Tat antibody was detected by double-antibody sandwich enzyme-linked immunosorbent assay. Two-way analysis of variance was used for data analysis, **P < 0.01, ***P < 0.001.

To further examine the toxicity *in vivo*, wild-type Tat-86 and Tat-R5M4 were injected intravenously into wild-type BABL/c mice at 40 mg/kg. Compared with the negative control, Tat-86 or Tat-R5M4 treatment showed no significant abnormality in various enzymes for liver and kidney functions (**Figure 5a**). No histological changes in different organs were detectable except for minor inflammatory cell infiltration in the lungs after treatment with wild-type Tat-86 (**Figure 5b**). These results suggested that Tat-R5M4 is safe and does not alter the physiological function of major organs.

The inflammatory cytokines induced by HIV-1 Tat play important roles in HIV-1 pathogenesis.^49^ We compared the effect of Tat-86 and Tat-R5M4 on the expression of IL-10, TNF-α, and IL-6. The monocytes were treated with Tat-86 or Tat-R5M4 proteins. Tat-R5M4 had a reduced ability to induce the expression of IL-10, TNF-α, or IL-6 (**Supplementary Figure S10**). Alternatively, it has been reported that Tat enhances the replication of Kaposi's sarcoma herpes virus.^50^ To examine whether Tat-R5M4 maintained this capability, BCBL-1 cells that were latently infected with KHSV were treated with Tat-86 or Tat-R5M4.^51^ Compared with Tat-86, Tat-R5M4 induced a much lower expression of viral proteins, including ORF45 and K8. Its ability to induce viral replication was also significantly reduced (**Supplementary Figure S11**). During the analysis for acute toxicity, we noticed that after injection of Tat-86, splenomegaly occurred in all the experimental mice, while the injection of Tat-R5M4 did not show any significant change (**Figure 5c**, **d**), suggesting that the immunogenicity of Tat-R5M4 was lower than that of Tat-86. To further examine the effect of mutations on immunogenicity, we immunized the wild-type mice with Tat-86 or Tat-R5M4 proteins and found that the mice immunized with Tat-R5M4 produced a significantly lower titer of anti-Tat antibodies than those immunized with Tat-86 (**Figure 5e**).

## Discussion

In this study, the Tat mutant Tat-R5M4 showed potent capability to activate latently infected CD4^+^ T lymphocytes. In the latent-infection state, the expression of both spliced and full-length mRNA of HIV-1 is inhibited, and insufficient endogenous Tat protein exists within the cells. If extra Tat protein is added, the P-TEFb complex and CBP/P300 would be specifically recruited to the HIV-1 promoter region. The transcription would restart, leading to the activation of the latent state. We have reported that a series of miRNAs interact with the 3ʹ termini of all HIV-1 mRNAs and inhibit mRNA expression of both spliced and full-length HIV-1. The expression of early-phase proteins like Tat is also affected by this mechanism.^13^ However, the purified Tat-R5M4 protein could avoid the suppression effect of these resting CD4^+^ T lymphocyte-enriched cellular miRNAs and directly activate latency, which is another advantage of our strategy. Furthermore, through various latency models, we always found that the combination of Tat-R5M4 with the HDACi SAHA exerted a significant increase in activation efficiency than the separate treatments. In the *in vitro* system, SAHA only activated about 31% of cells, while Tat-R5M4 activated a relatively higher proportion. When both reagents were combined, the activation efficiency reached 76% or more. It has been surmised that the HDACi is not an ideal reagent to activate viral latency when used alone.^18^ However, when combined with Tat-R5M4, a significant synergistic effect occurred in the various latency models used in our experiments. A reasonable explanation is that SAHA promotes histone acetylation in the region of the HIV-1 promoter, turning this region into a transcriptionally active site that is more accessible for Tat to specifically bind to the transactivation response region and recruit many more transcriptional factors.

Tat has been demonstrated to cause severe cytopathic effects and to contribute to HIV-1 pathogenesis. Tat induces apoptosis in bystander cells, modulates the expression of various genes to cause immune suppression, and induces and activates the replication of Kaposi's sarcoma herpes virus.^29,50,52,53^ However, the safety of Tat protein has been proved by clinical trials in several Tat vaccine studies in which the bioactive recombinant wild-type Tat-101 was used.^32,33,34,35,43^ We examined the acute toxicity of Tat-R5M4 in mice by directly injecting the recombinant protein intravenously. A dosage as high as 40 mg/kg caused no pathological alteration in mice, indicating that Tat-R5M4 was safe for mice in a short duration. However, long-term toxicity remains to be examined. We also found that Tat-R5M4 induced much less expression of immune-suppressive cytokines and had a reduced capability to stimulate the replication of Kaposi's sarcoma herpes virus. Moreover, its immunogenicity was much lower than that of wild-type Tat. Because of these effective mutations, we believe that Tat-R5M4 merits further development for clinical use. In addition, we also believe that bioactive recombinant wild-type Tat-101, after undergoes mutations to decrease the toxicity but keep the immunogenicity, could be further developed as a safer vaccine.

To develop a potent LRA for the functional cure of HIV-1 infection with the “shock and kill” strategy, we adapted the logic of the virus itself and generated a recombinant Tat-R5M4 that exerts its specificity and efficiency for latency activation and also shows great potential to become a protein drug, especially when used in combination with HDACi. Importantly, genetic diversity analysis indicated that compared to other mutants, Tat-R5M4 activates a larger number HIV-1 proviruses hiding at various chromosomal locations. The activation by Tat mutants could therefore reshape the quasispecies spectrum activated by HIV-1 latency.^54^ Furthermore, on the basis of our data, we propose that the strategy to manipulate Tat protein itself or its related partners/pathways could open new avenues to develop new types of LRAs that will play an important role in HIV treatment.

## Materials and Methods

***Ethic statements.*** This research was approved by the ethics review board of Sun Yet-sen University and Guangzhou 8th People's Hospital. The written informed consent was provided by study participants.

***Plasmid constructions.*** HIV-1 *tat-86* gene was amplified from pLSN-Tat and site mutations in *tat-86* were performed as previously described.^55^ *tat-86* and its mutants were then inserted into pcDNA3.1 or pET28a vectors. The *Bcl-2* gene was subcloned into the *nef* region of pNL4/3-▵E-*gfp*. Its reading frame is consistent with the beginning of *nef*. The new construct was then named as pNL4/3-E-*gfp-bcl-2*.

***Protein expression, purification, and characterization.*** *Escherichia coli* cells (10 g wet weight) expressing Tat-86 or Tat-R5M4 were sonicated in 40 ml of lysis buffer (20 mmol/l sodium phosphate, pH 7.8, 2.5% glycerol, 0.2 mmol/l phenylmethylsulfonyl fluoride, 5 mmol/l Dithiothreitol, 50 mmol/l mannitol, 10 mmol/l ascorbic acid, and 500 mmol/l NaCl) using an ultrasonic liquid processor (Model VCX150, Sonics) with three 20-second bursts. The lysate was then clarified by centrifugation at 12,000 g for 30 minutes and purified by heparin-agarose chromatography and subsequently ion exchange.^56,57^ The purities of expressed proteins were >95%. To remove endotoxin, 1% Triton X-114 was added to the protein preparation. The mixture was incubated at 4 °C for 30 minutes and then incubated at 37 °C for 10 minutes, followed by centrifugation at room temperature. The upper aqueous phase was carefully removed and subjected to Triton X-114 phase separation for at least two times.^58^ The final concentration of endotoxin was analyzed with tachypleus amebocyte lysate and proved to be less than 0.5 EU/ml. If necessary, the purified proteins were labeled with NHS-rhodamine labeling kit (Thermo).

***Analysis of transactivation activity.*** The TZM-b1 cells were transfected with 100 ng pcDNA3.1-Tat-86, pcDNA3.1-Tat-R5M4, or other control plasmids respectively. Alternatively, TZM-b1 cells were directly treated with purified Tat-86 or Tat-R5M4 proteins at various concentrations. The cells were then washed and collected after 48 hours. The luciferase assay was performed as described previously.^59^

***Apoptosis assay.*** The CD4^+^ T cells were cocultured with purified Tat-86 or Tat-R5M4 proteins for 2 days, then washed twice with cold phosphate-buffered saline buffer and stained with annexinV-PE and 7AAD. The ratio of cells undergoing apoptosis was analyzed by FACS.

***MTS (3-(4,5-diethylthiazol-2-***...(4-sulfo phenyl)-2H-etrazolium),inner salt) assay. The Jurkat cells were cultured at a density of 5 × 10^4^ cells per well in 96-well cell culture plates and treated with purified Tat-86 or Tat-R5M4 proteins. After 2 days, the cell titer 96 aqueous one solution reagent (Promega, Madison, WI) was added to each well according to the instructions of manufacturer. After treatment for 2 hours, the cell viability was determined by measuring the absorbance at 493 nm.

***Generation of latently-infected primary CD4+ T lymphocytes* in vitro.** The pNL4/3-E-*gfp-bcl-2* and pMD-G were cotransfected to HEK293T cells to generate HIV-1/VSV-G pseudoviruses. In parallel, the primary CD4^+^ T-lymphocytes were isolated from peripheral blood mononuclear cells of healthy donors and activated with 1μg/ml anti-CD3 and 2 µg/ml anti-CD28 antibodies. They were then infected with pseudoviruses. The GFP-positive cells were sorted out and expanded with anti-CD3 and anti-CD28 antibodies, plus IL-2 at 100 U/ml for 7 days. After culture for 2–3 weeks with eventually lower concentrations of IL-2, the culture was maintained without IL-2 for one more week. The GFP-negative cells with more than 99.9% purity were isolated using FACS and subjected to the activation by various reagents.

***Latency activation in primary CD4+ T-lymphocytes isolated from HIV-1-infected individuals receiving ART.*** HIV-1-infected individuals who had the viral particles in the blood plasma less than 20 copies/ml and had the number of CD4^+^ lymphocytes higher than 200 per ml were recruited for our study. The existence of HIV-1 proviruses in the CD4 T-lymphocytes was analyzed by Alu-PCR and cytokine stimulation was performed as previously described.^13^ The resting CD4^+^ T lymphocytes were added with 25 ng/ml PMA/1 mg/ml ionomycin, 335 nmol/l SAHA, 1 µmol/l Tat-R5M4 or 335 nmol/l SAHA plus 1 µmol/l Tat-R5M4 and viral RNAs in culture supernatant were quantitated by real-time reverse transcription PCR at 48 hours later. For detecting replication-competent viruses, the cells were exposed to 200Gy X-ray irradiation and then cocultured with the freshly activated CD4^+^ T-lymphocytes from healthy donors. Viral replication was then monitored by detecting HIV-1 p24 in the supernatant of cell culture.

***Genetic diversity analysis of activated HIV-1 viruses.*** The genetic diversity of HIV-1 quasispecies under different reagent treatments was assessed by sequencing the *env* V1-V3 region with the primer pairs described previously.^23^ To minimize the sampling bias, single genome amplification method was performed and 30 independent PCR products obtained from each sample were used for cloning.^60^ The alignments of HIV-1 sequences were built by using MUSCLE.^61^ All ambiguous positions were removed for each sequence pair. After that, the average genetic distance between one given clone and the relevant entire population in each sample was calculated using MEGA 6. Moreover, the two-tailed Mann-Whitney *U*-test was conducted with Prism 5.0 software (www.graphpad.com) for comparing the genetic diversities between different samples. To further depict the globe landscape of HIV diversity, the phylogenetic bootstrap consensus trees were also constructed using neighbor-joining method with 1,000 bootstrap replications implemented in MEGA 6.

**SUPPLEMENTARY MATERIAL** **Table S1.** Mutation Round1-3 and Detection of Transactivation Activity. **Table S2.** Mutation Round 4–6 and Detection of Transactivation Activity and Apoptosis. **Figure S1.** Transactivation activity of HIV-1 Tat and Tat mutants. **Figure S2.** Purification of Tat-86, Tat-R4M4, R4M5, R4M7 and R5M4. **Figure S3.** Transactivation activities of various Tat mutants. **Figure S4.** Transactivation activity of purified Tat protein in TZM-bl cells. **Figure S5.** Rhodamine labeled Tat-R5M4 showed capability to enter the cells in a dose-dependent manner. **Figure S6.** Transmembrane activity of Tat-86 and Tat-R5M4 in vitro. **Figure S7.** Transmembrane ability of Tat-R5M4 in vivo. **Figure S8.** Generation of latency model in vitro. **Figure S9.** Activation of the latently-infected CD4+ T-lymphocytes from HIV-1-infected individuals receiving suppressive ART by Tat-R5M4. **Figure S10.** Tat-R5M4 reduced the ability of Tat to induce cytokine secretions. **Figure S11.** Tat-R5M4 reduced the ability of Tat-86 to induce the replication and expression of Kaposi's sarcoma herpes virus (KSHV).

## Author Contributions

G.N.G. and H.Z. designed the experiments; G.N.G. performed the most experiments, B.F.L constructed most of the plasmids and performed the screening of Tat mutants; C.C., J.L.(i) participated in generating the in vitro latency model; K.W. performed some experiments including mouse feeding and tissue sections; T.P., J.S.Z., F.H., F.Y., J.L.(i) performed some of the western blotting experiments. E.S.K analyzed the influence of Tat or Tat-R5M4 on KSHV replication; X.C.M. performed some experiments on sequencing of HIV-1 quasispecies; J.L.(iu) performed the bioinformatics analyses; Y.Y., J.Z.(hou)., C.L. participated in analyzing the data. W.P.C recruited the patient samples. H.Z. supervised the whole research and interpreted the data, G.N.G and H.Z. wrote the manuscript.
