# Supplementary material for: Development of an Attenuated Tat Protein as a Highly-effective Agent to Specifically Activate HIV-1 Latency
Source: Mol Ther. 2016 Jul 19;24(9):1528–37. doi: 10.1038/mt.2016.117 (PMC5113098; doi:10.1038/mt.2016.117)
Supplement: Supplementary Figures and Tables [file mt2016117x1.pdf]

Round1 Mutations		Transactivation Activity	Round2 Mutations		Transactivation Activity
R1M1	M22	−/−	R2M1	M8,10	−/−
R1M2	M23	+/+	R2M2	M11,12	−/−
R1M3	M36	+/+	R2M3	M22,23	+/−
R1M4	M39	+/+	R2M4	M28,29	−/−
R1M5	M51	+/+	R2M5	M46,47	−/−
R1M6	M60	−/−	R2M6	M36,39	+/+
R1M7	M61	−/−	R2M7	M66,67	+/+
R1M8	M62	−/−	R2M8	M66,68	+/+
R1M9	M63	−/−	R2M9	M66,69	+/+
R1M10	M64	−/−	R2M10	M67,68	+/+
R1M11	M65	−/−	R2M11	M67,69	+/+
R1M12	M66	+/+	R2M12	M68,69	+/+
R1M13	M67	+/+			
R1M14	M68	+/+	Round3 Mutations		
R1M15	M69	+/+	R3M1	M66,67,68	+/+
R1M16	M70	−/−	R3M2	M66,67,69	+/+
R1M17	M71	−/−	R3M3	M66,68,69	+/+
R1M18	M72	−/−	R3M4	M67,68,69	+/+

Supplementary Table1:

Mutation Round1-3 and Detection of Transactivation Activity

+/+, >70% wild type; +/- , > 20% wild type activity; -/- , 20% or <20% wild type activity.

Amino acids in mutation sites are all turned to Ala(gca).

Round4 Mutations		Transactivation activity	Apoptosis	Round5 Mutations		Transactivation activity	Apoptosis
R4M1	M28,29,46,47	−/−	ND	R5M1	M36,39,66,67,68	−/−	−/−
R4M2	M22,23,46,47	−/−	ND	R5M2	M36,66,67,68, 77	+/−	−/−
R4M3	M66,67,68,69	−/−	ND	R5M3	M51,36,66,67,68	+/−	−/−
R4M4	M36,66,67,68	+/−	−/−	R5M4	M36,66,67,68,77	+/+	−/−
R4M5	M39,66,67,68	+/+	−/−	Round6 Mutations		Transactivation activity	Apoptosis
R4M6	M51,66,67,68	+/−	ND	R6M1	M51,77,36,66,67,68	−/−	ND
R4M7	M77,66,67,68	+/+	−/−	R6M2	M51,77,39,66,67,68	−/−	ND
R4M8	M36,67,68,69	−/−	ND	R6M3	M51,36,39,66,67,68	+/−	ND
R4M9	M39,66,68,81	−/−	ND	R6M4	M36,51,66,67,68,77	+/−	ND
R4M10	M51,39,66,68	−/−	ND	R6M5	M39,51,66,67,68,77	−/−	ND

Supplementary Table2:

Mutation Round 4-6 and Detection of Transactivation Activity and Apoptosis

+/+, >70% wild type; +/- , > 20% wild type activity; -/- , 20% or <20% wild type activity.

Amino acids in mutation sites are all turned to Ala(gca).

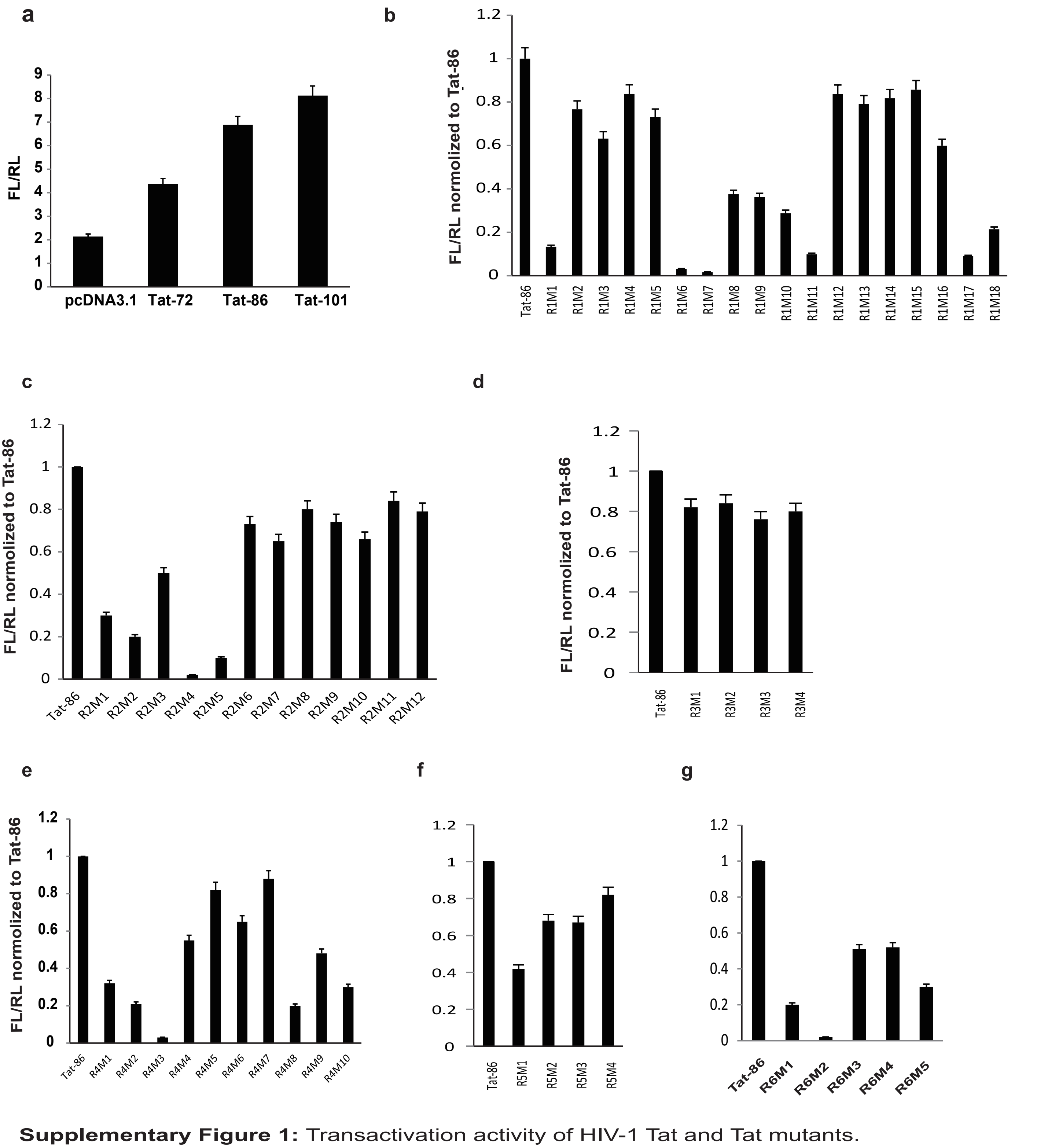

Supplementary Figure 1: Transactivation activity of HIV-1 Tat and Tat mutants. (a)TZM-bl cells were co-transfected with Relina and 100ng of pcDNA3.1, pcDNA3.1-Tat-72, pcDNA3.1-Tat-86 or pcDNA3.1-Tat-101.Ratio of Firefly Luciferase/Relina Luciferase (FL/RLratio) was analyzed after 48 hours.(b-g)Transactivation activity of products of Mutation Round1-6 were analyzed and normalized to Tat-86.

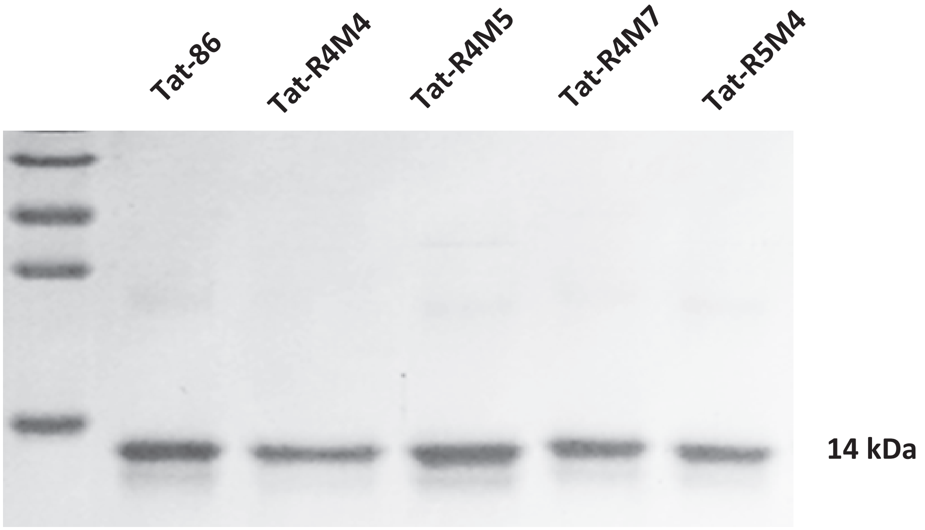

Supplementary Figure 2 : Purification of Tat-86, Tat-R4M4, R4M5, R4M7 and R5M4. The proteins were expressed in DE3 E.Coli and purified through heparin-agarose chromatography and subsequent ion exchange chromatography. After eliminating endotoxin, the proteins were subjected to SDS-PAGE.

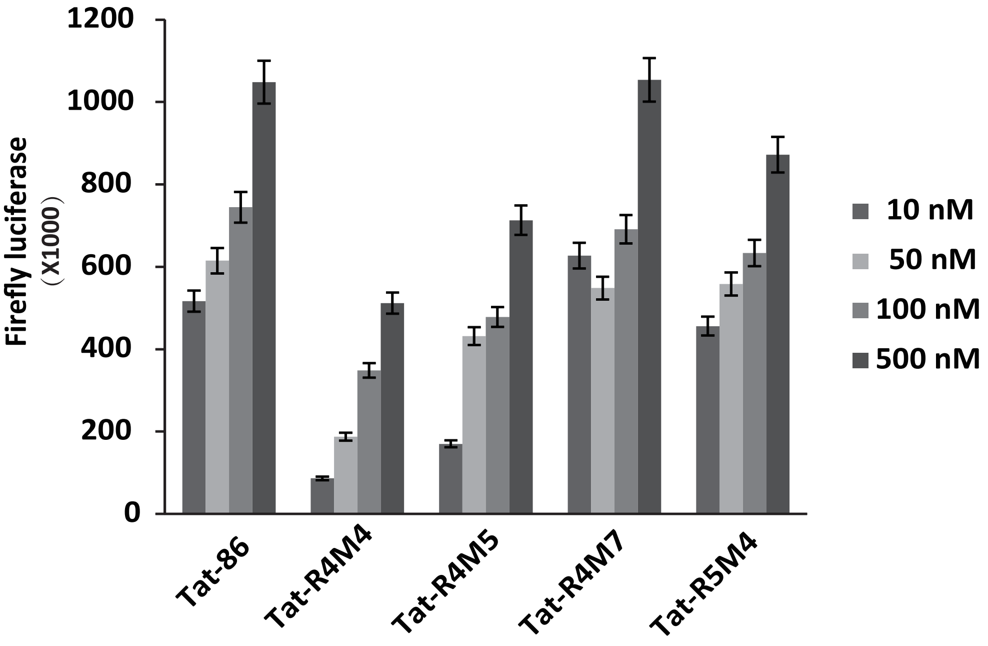

Supplementary Figure 3: Transactivation activities of various Tat mutants. The TZM-bl cells were co-cultured with Tat-R4M4, Tat-R4M5, Tat-R4M7, or Tat-R5M4 recombinant proteins at the concertrations of 10 nM, 50 nM, 100 nM, or 500 nM, the luciferase activity was analyzed after 2 days.

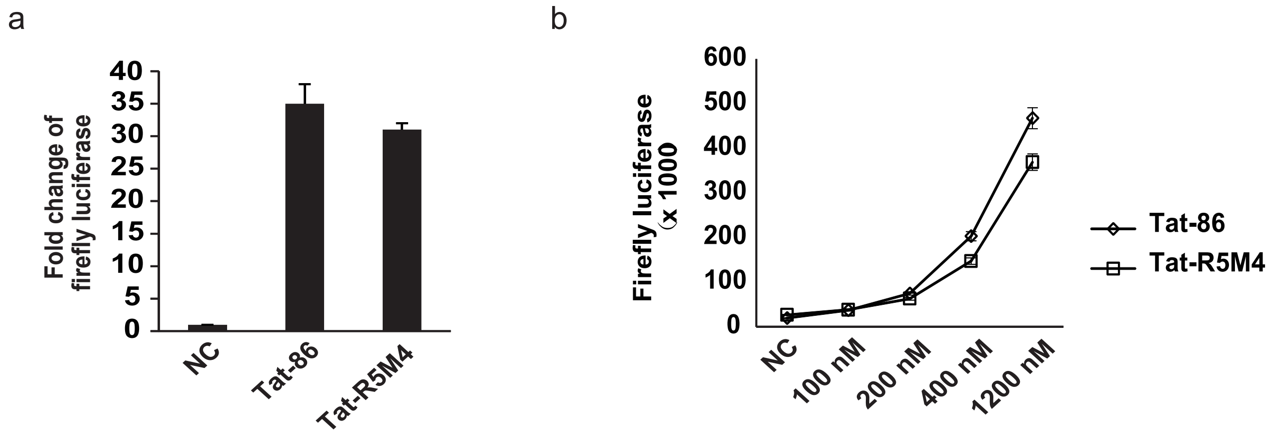

Supplementary Figure 4: Transactivation activity of purified Tat protein in TZM-bl cells. (a)500nM Purified Tat-86 or Tat-R5M4 proteins were added into culture medium of TZM-bl cells, and Firefly Luciferase activity was analyzed after 2 days. (b) a dose-dependent experiment was performed.

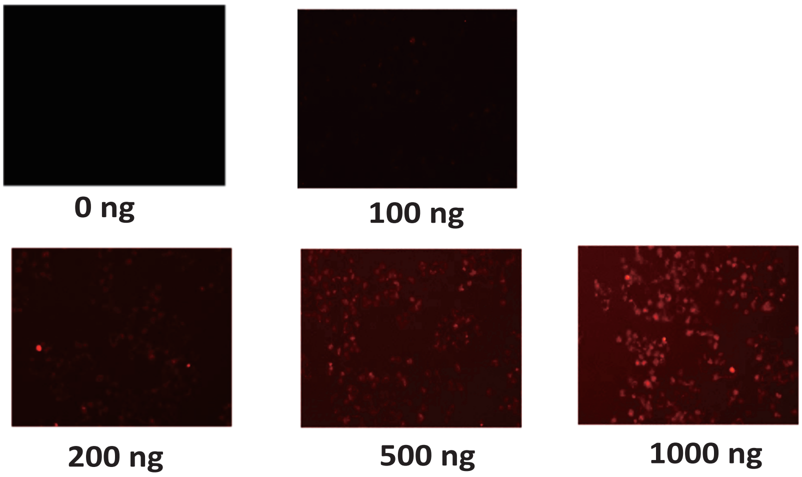

Supplementary Figure 5: Rhodamine-labeled Tat-R5M4 showed capability to enter the cells in a dose-depent manner. Tat-R5M4 was labeled with NHS-rhodamine and added to the supernatant of TZM-bl cells. Cells were washed with PBS after 6 hours and the distribution of fluorescence was observed with fluorescence microscopy.

a

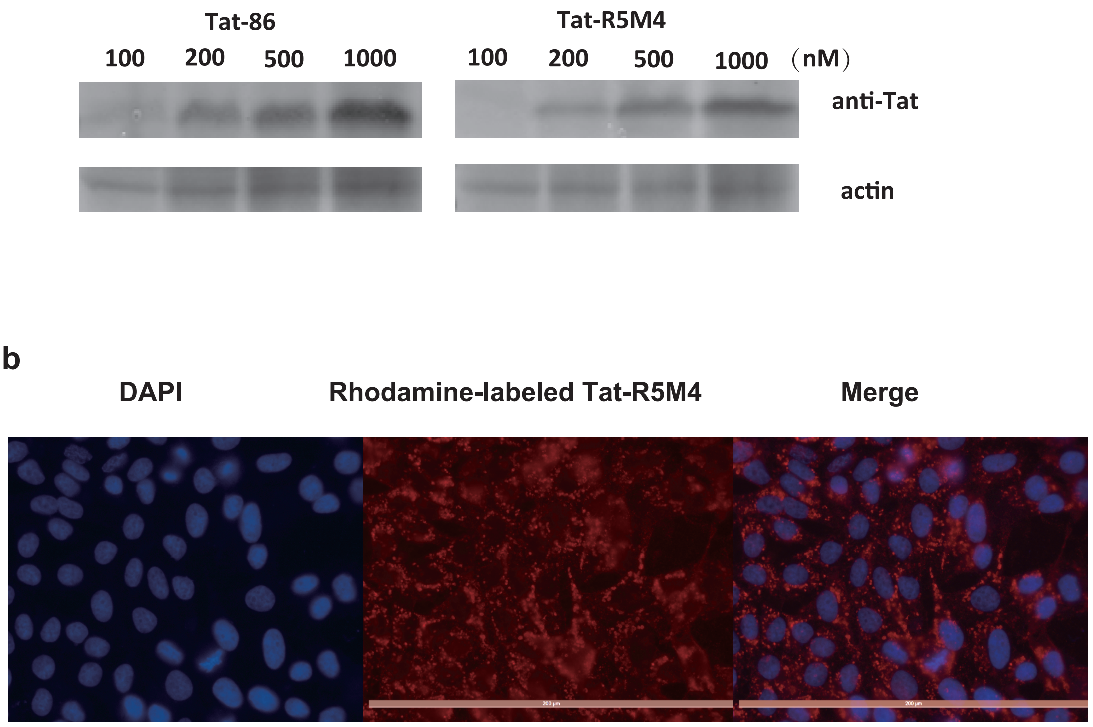

Supplementary Figure 6: Transmembrane activity of Tat-86 and Tat-R5M4 *in vitro.* (a)TZM-bl cells were co-cultured with Tat-86 or Tat-R5M4 at the concentration of 100nM, 200nM, 500nM or 1000nM. After 2 days, cells were washed and lysed. Western blotting was performed with monoclonal anti-Tat antibody. (b)TZM-bl cells were seeded onto 35-mm glass-bottom culture dishes(MatTek) and treated with 500nM Rhodamine-labeled Tat-R5M4 for 6 hours. Cells were washed and stained with DAPI, and observed with fluorescence microscope.

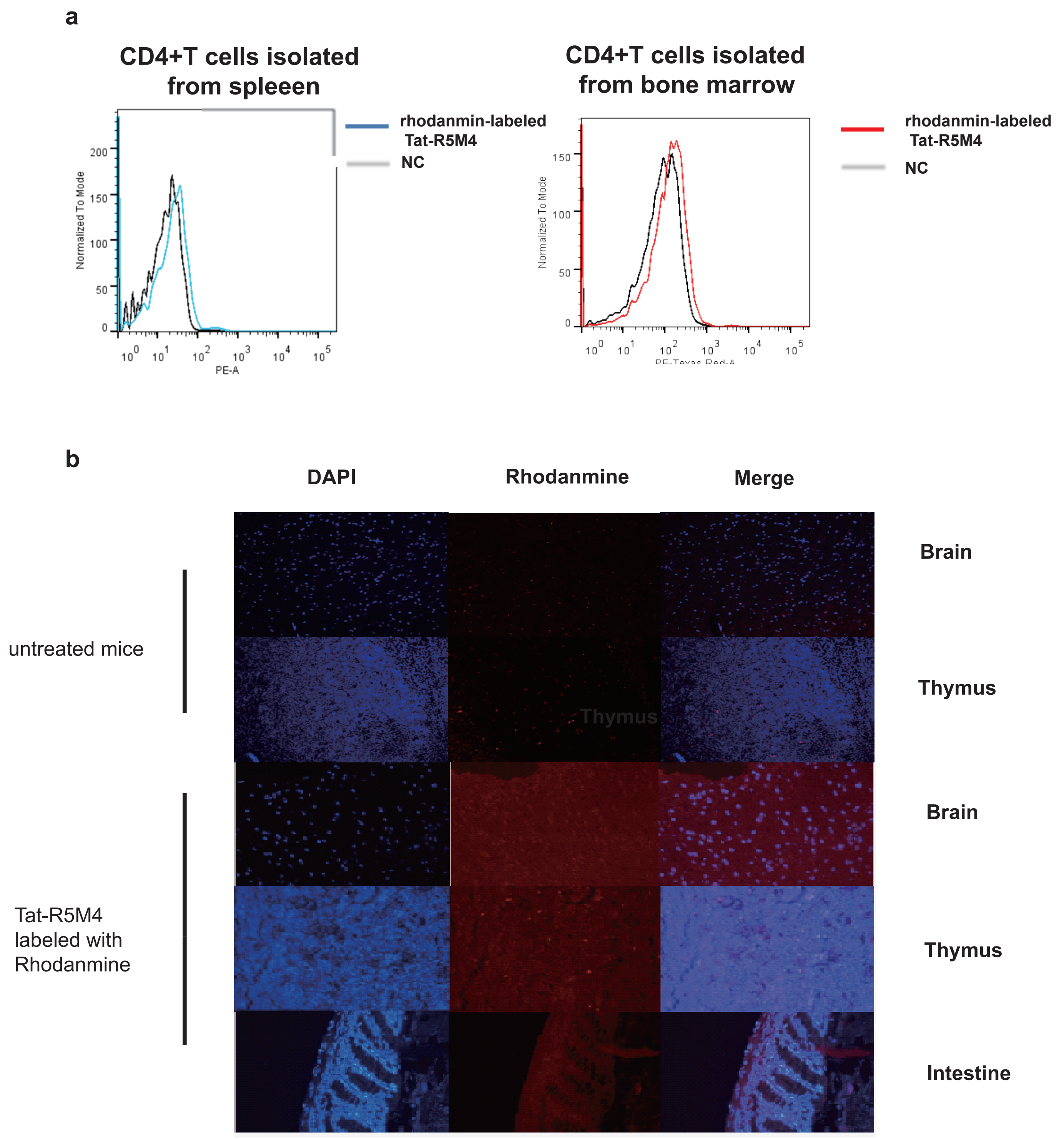

Supplementary Figure 7: Transmembrane ability of Tat-R5M4 *in vivo.*
Tat-R5M4 was labeled with NHS-rhodamine and intravenously injected into BABL/c mice.(a) The CD4+ T-lymphocytes from both spleen and bone marrow were isolated and analyzed by FACS. (b)Brain, thymus and intestine were dissected, stained with DAPI and subjected to fluorescence microscopy analysis.

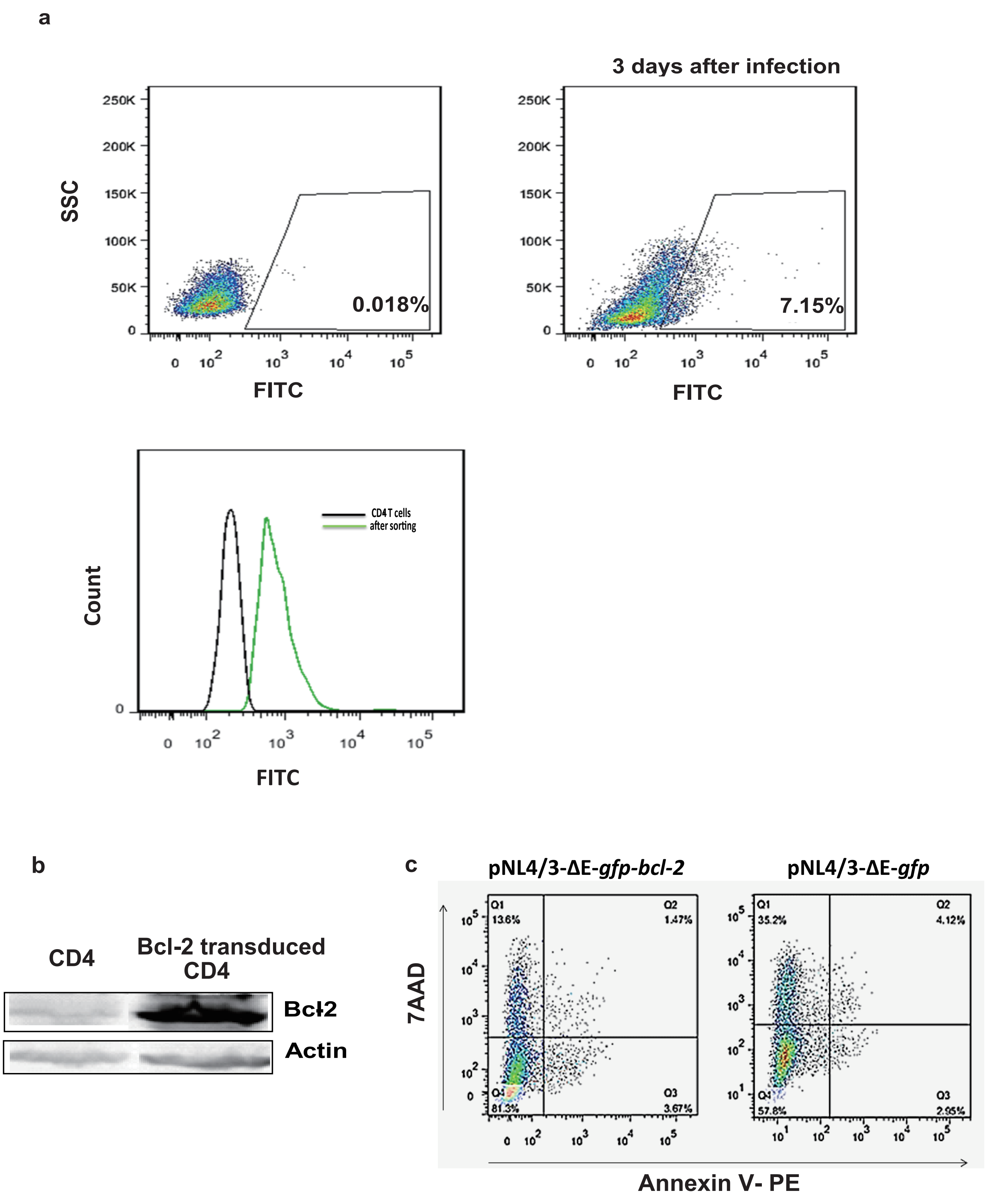

Supplementary Figure 8

d

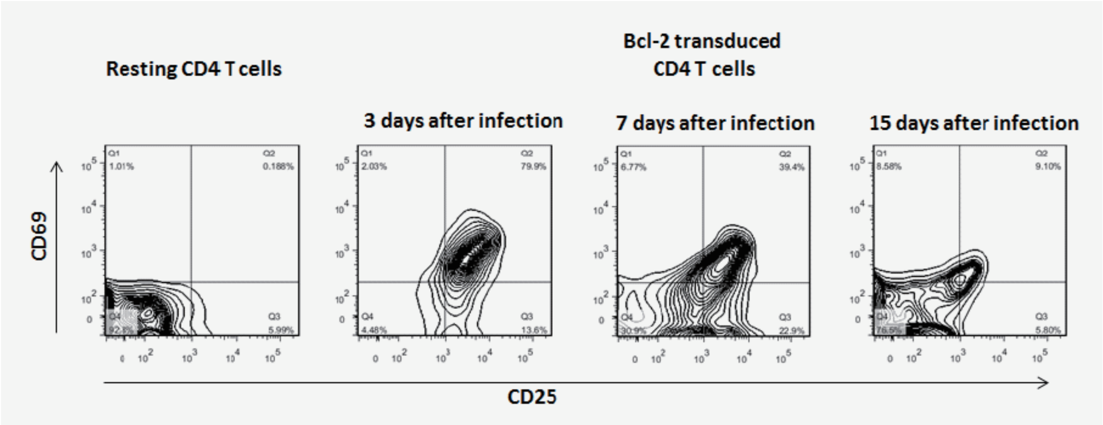

e

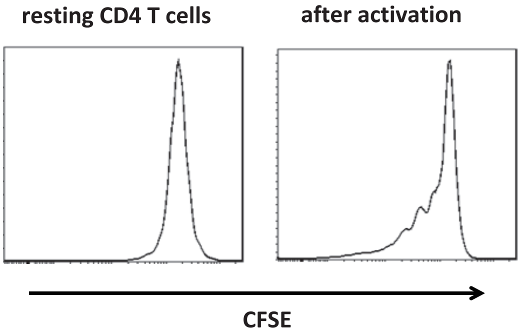

f

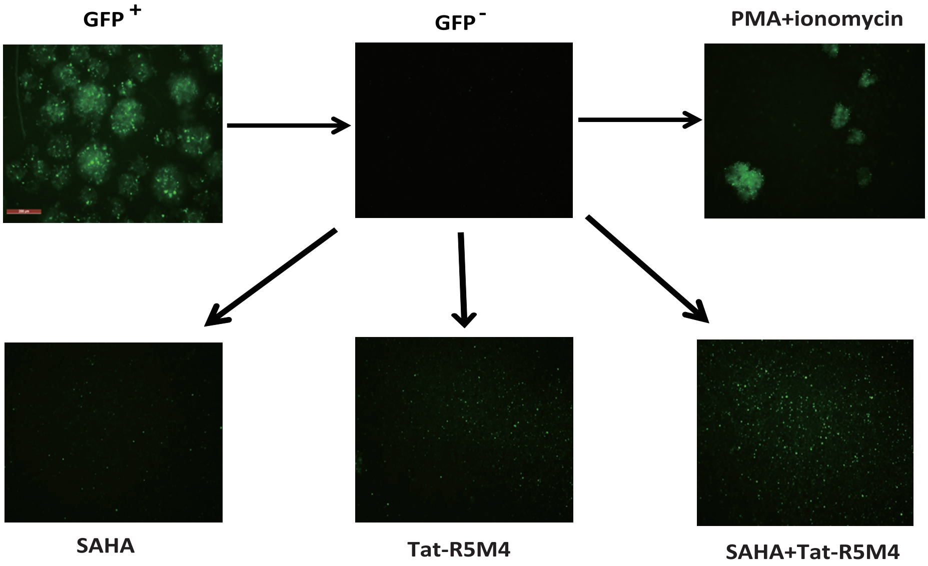

Supplementary Figure 8: The generation of latency model in vitro. The activated CD4$^+$ T lymphocytes were infected with HIV-1/VSV pseudoviruses.(a) GFP-positive cells were sorted out and lysed, (b) western blotting was performed with anti-Bcl-2 antibody. (c) Proportions of apoptosis were compared between the cells infeted with pseudovirus with or without Bcl-2 expression. (d) The resting state of latently infected CD4$^+$T cells were analyzed with FACS. (e) Proliferation of latently infected CD4$^+$ T cells after activation. (f)Activation of latently infected CD4$^+$T cells with PMA/ionomycin, SAHA, Tat-R5M4 or SAHA plus Tat-R5M4. Two days after activation, green fluorescence was observed with fluorescence microscopy.

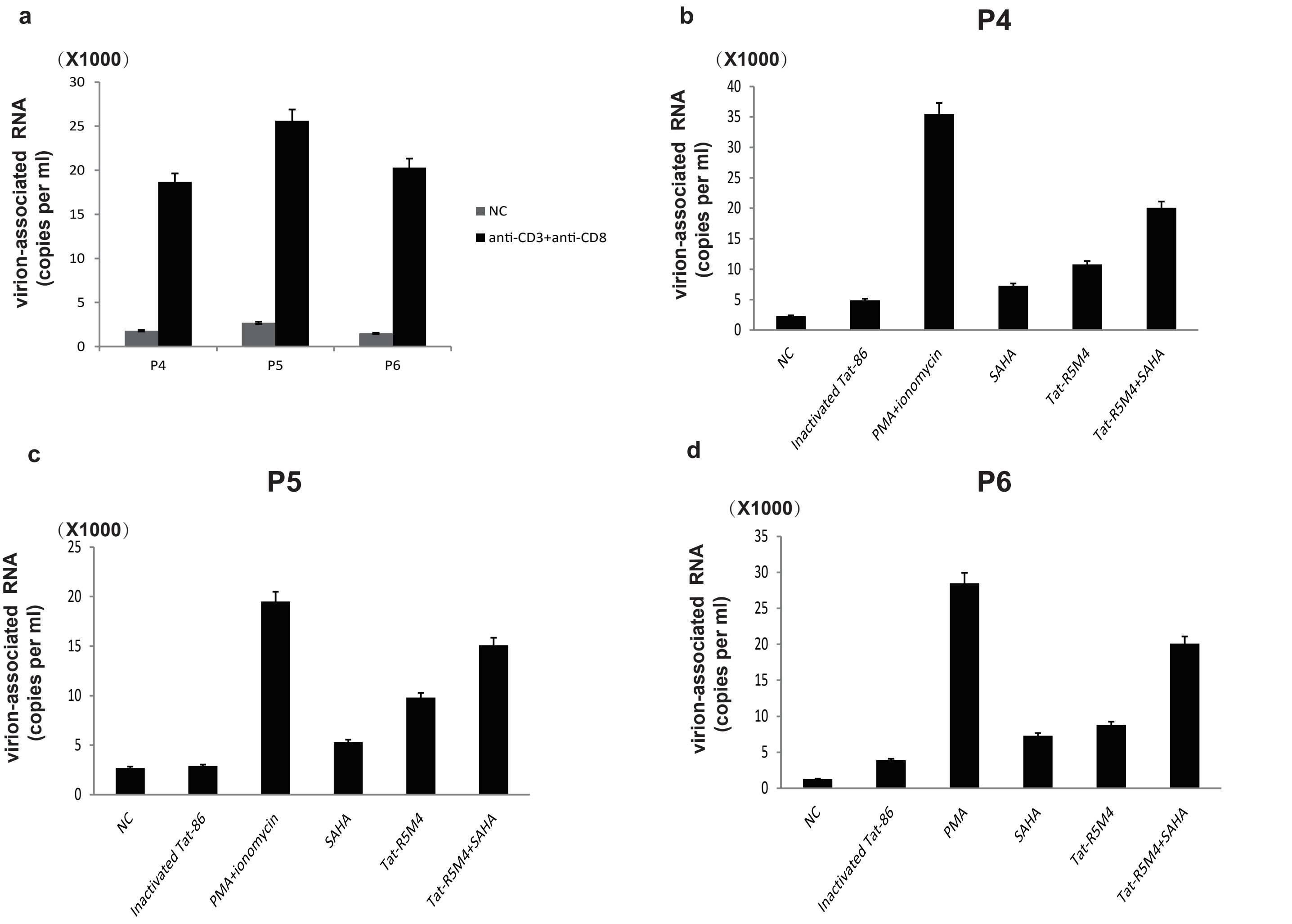

Supplementary Figure 9: Activation of the latently-infected CD4+ T-lymphocytes from HIV-1-infected individuals receiving suppressive ART by Tat-R5M4. (a) CD4+ T-lymphocytes from patients 4-6 were isolated and activated by anti-CD3 and anti-CD28 antibodies. (b,c,d)The CD4+ T-lymphocytes isolated from HIV-1-infected individuals were cultured in the RPMI1640 conditioned medium and activated with PMA plus ionomycin, inactivated Tat, SAHA, Tat-R5M4, or SAHA plus Tat-R5M4. After 48 h, the viral particles in supernatant were harvested and viral RNA was extracted and quantitatively analyzed by real-time RT-PCR.

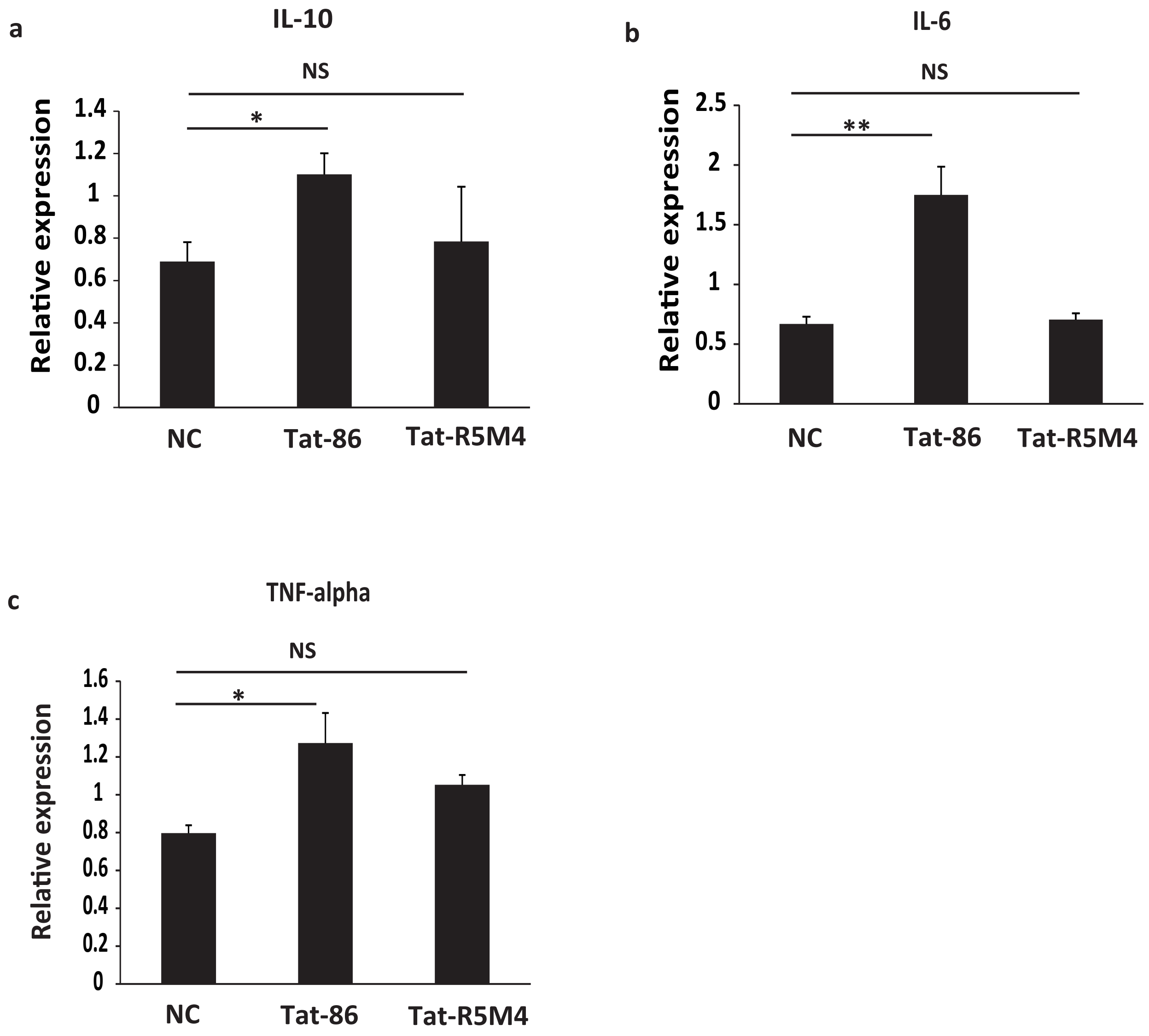

Supplementary Figure 10:Tat-R5M4 had the lower ability than wild-type Tat to induce the secretion of cytokines. The CD14-positive monocytes were isolated from healthy donors and then co-cultured with purified Tat-86 or Tat-R5M4 for 6 hours. Total RNA was extracted with Trizol reagent and reverse transcribed. The transcriptional expression of IL-10, IL-6 and TNF-alpha were analyzed with real-time RT-PCR. *$p < 0.05$, ** $p < 0.01$, NS, not significant in a paired *t* test.

a

b

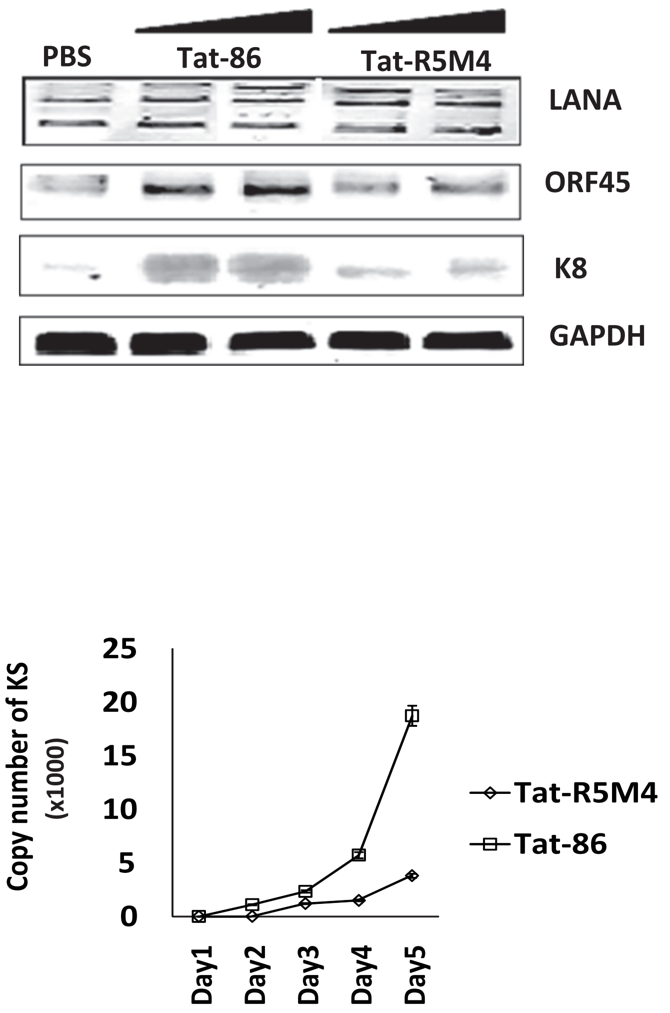

Supplementary Figure 11:Tat -R5M4 reduced the ability of Tat-86 to induce the replication and expression of Kaposi's sarcoma herpes virus(KSHV). Bcbl-1 cells were treated with Tat-86 or Tat-R5M4 for 5 days. (a) Expression of LANA,ORF45 and K8 were analyzed with western blotting, and (b) the amount of KSHV DNA in the supernatant was analyzed with real-time PCR.
